# Evaluation of hepatocyte-derived microRNA-122 for diagnosis of acute and chronic hepatitis of dogs

**DOI:** 10.14202/vetworld.2018.667-673

**Published:** 2018-05-21

**Authors:** S. R. Eman, A. A. Kubesy, T. A. Baraka, F. A. Torad, I. S. Shaymaa, Faten F. Mohammed

**Affiliations:** 1 Department of Internal Medicine and Infectious Diseases, Faculty of Veterinary Medicine, Cairo University, Giza 12211, Egypt; 2 Department of Surgery and Anesthesia, Faculty of Veterinary Medicine, Cairo University, Giza 12211, Egypt; 3 Department of Clinical Pathology, Faculty of Veterinary Medicine, Cairo University, Giza 12211, Egypt; 4 Department of Pathology, Faculty of Veterinary Medicine, Cairo University, Giza 12211, Egypt

**Keywords:** canine, cytology and histopathology, hepatitis, hepatocyte derived miRNA-122, ultrasonography

## Abstract

**Aim:**

This study was performed to evaluate the diagnostic value of hepatocyte-derived microRNA (miRNA)-122 in acute and chronic hepatitis of dogs.

**Materials and Methods:**

A total of 26 dogs presented at Veterinary Teaching Hospital, Faculty of Veterinary Medicine, Cairo University, 16 dogs out of 26 showing clinical signs of hepatic insufficiency were subjected to clinical, ultrasonographic, hematobiochemical and ultrasound-guided fine-needle biopsy for cytological and histopathological investigations. On the basis of these results, 7 dogs out of 16 dogs were found to be suffering from acute hepatitis and 9 dogs suffering from chronic hepatitis. 10 clinically healthy dogs were kept as control. Serum hepatocyte-derived miRNA-122 was analyzed by real-time quantitative polymerase chain reaction in all dogs.

**Results:**

The dogs suffering from acute hepatitis manifested jaundice, vomiting, and depression while dogs with chronic hepatitis manifested anorexia, abdominal distension, weight loss, and melena. Hematological parameters showed normocytic normochromic anemia and thrombocytopenia in both acute and chronic hepatitis groups. Alanine aminotransferase (ALT), aspartate aminotransferase (AST), alkaline phosphatase (ALP), and total bilirubin were significantly higher than control values in acute hepatitis. In chronic hepatitis, total protein and albumin were significantly lower than control values with normal ALT, AST, ALP, and gamma-glutamyltransferase values. Ultrasonography revealed a diffuse decrease in hepatic echogenicity in acute hepatitis while the increase in hepatic echogenicity and anechoic ascetic fluid in chronic hepatitis. Cytology revealed hepatic vacuolar degeneration and histopathology revealed necrosis and apoptosis of hepatocyte in acute hepatitis while revealed massive fibrous tissue proliferation in hepatic parenchyma in chronic hepatitis. Serum miRNA-122 analysis, normalized for glyceraldehyde-3-phosphate dehydrogenase expression revealed a significant increase in acute hepatitis accompanied with elevation in ALT and AST, while in chronic hepatitis, elevation of serum miRNA-122 was accompanied with ALT and AST of the normal range.

**Conclusion:**

Serum hepatocyte-derived miRNA-122 is of diagnostic value and highly stable blood indicator for the detection of hepatocellular injury in dogs than aminotransferases, especially in cases where aminotransferases do not exceed normal serum level.

## Introduction

Primary hepatitis (PH) is the most occurring liver disease in dogs. Acute and chronic hepatitis are the most common forms, and chronic form may be associated with cirrhosis or not. PH represents 0.5% of the canine referral population with the high prevalence of chronic form [1]. Labrador Retrievers, Doberman Pinschers, English Cocker Spaniels, Bedlington Terriers, West Highland White Terriers, and many other breeds are the most susceptible breeds [2].

Liver diseases usually associated with non-specific clinical signs including depression, lethargy, anorexia, vomiting, diarrhea, and weight loss as well as characteristic clinical signs including jaundice, bleeding tendency, and ascites [3].

Diagnosis of liver diseases is considered a challenge for a veterinarian as specific clinical signs become overt only when hepatocellular damage became massive. Hence, it requires a lot of diagnostic workup including clinical examination, ultrasonography, and laboratory testing as well as cytology and/or histopathology [4].

Dogs with liver disease remain subclinical for a long period. In addition, these subclinical animals often show normal enzymatic levels so that they are difficult to be diagnosed by current screening[5]. New hepatic diagnostic biomarkers are needed to overcome this lack in sensitivity of current screening methods [6].

Recently, hepatocyte-derived microRNAs (miRNAs) became stable and sensitive diagnostic blood biomarkers for liver illnesses in human and animal models [7-10].

miRNAs are cluster of small non-coding RNAs that play vital roles in hepatic functions and imperative regulators of post-transcriptional gene expression [11]. miRNA-122 is a specific indicator of hepatocellular injury as it represents 72% of all miRNAs population in the liver [12].

The aim of this study was to evaluate the efficiency of hepatocyte-derived miRNA-122 for diagnosing acute and chronic hepatitis in dogs.

## Materials and Methods

### Ethical approval and informed consent

The study was approved by the Institutional Animal Care and Use Committee, Cairo University (CU II F 44 17). All owners were aware that their dogs will be used for research purposes and signed consent indicating their approval.

### Animals

The study was conducted on 26 dogs admitted to Veterinary Teaching Hospital, Faculty of Veterinary Medicine, Cairo University. All dogs underwent comprehensive clinical examinations, ultrasonography, laboratory analysis, and cytology and/or histopathology of the liver. On the basis of such examinations, dogs were categorized into Group A (n=7), dogs diagnosed to have acute hepatitis; Group B (n=9), dogs diagnosed to have chronic hepatitis and one case was died and exposed to histopathological examination in the second group; and a control group (n=10), dogs admitted to hospital for annual checkup with normal hematological, parasitological, biochemical, and ultrasonographic (USG) investigations.

### Physical examination

Complete physical examination included evaluation of vital signs (rectal temperature, pulse, respiration, lymph nodes, and mucous membranes), general condition, presence or absence of abdominal pain, and/or ascitic fluid.

### USG examination

Transabdominal ultrasonography was performed for the evaluation of hepatic parenchyma, hepatic blood vessels, biliary system as well as gallbladder through using 3.5-5 MHz convex probe according to previously described method [13].

### Hematology, serum biochemistry, and hepatocyte-derived miRNA-122

Blood samples were collected from all dogs for hematological examination by automated hematology analyzer, and serum was separated for the estimation of serum alanine aminotransferase (ALT), aspartate aminotransferase (AST), alkaline phosphatase (ALP), gamma-glutamyltransferase (GGT), total protein, albumin, and total bilirubin using specific kits according to manufacturer instructions (Spectrum Diagnostic Kits, Egypt).

Serum samples were stored at −20 till hepatocyte-derived miRNAs-122 was analyzed [6,14]. Briefly, RNA from samples was extracted using RNeasy Mini Kit (Catalogue No. 74104, QIAGEN, Germany) according to manufacturer instruction. Quantitative polymerase chain reaction (PCR) for the detection of hepatocyte-derived miRNA-122 was done using Quantitect SYBR Green PCR Kit (QIAGEN, Germany, Cat. No. 204141), oligonucleotide primers and probes used in SYBR Green Real-time PCR are shown in Table-1 [6,14], and cycling conditions for SYBR Green Real-time PCR are depicted in Table-2. Amplification curves and cycle threshold (CT) values were determined by the Stratagene MX3005P software. To estimate the variation of gene expression on the RNA of the different samples, the CT of each sample was compared with that of the control group [15].

**Table-1:** Oligonucleotide primers and probes used in SYBR Green Real-time PCR.

Gene	Primer sequence (5’-3’)	Reference
Dog GAPDH	GTCCCCACCCCCAATGTATC	[14]
	CTCCGATGCCTGCTTCACTACCTT	
MiR-122	Gcgagcacagaattaatacgac	[6]
	Tggagtgtgacaatggtgtttg	

PCR=Polymerase chain reaction, GAPDH=Glyceraldehyde-3-phosphate dehydrogenase

**Table-2:** Cycling conditions for SYBR Green Real-time PCR according to Quantitect SYBR Green PCR Kit.

Gene	GAPDH	MiR-122
Reverse transcription	50°C 30 min	50°C 30 min
Primary denaturation	94°C 5 min	94°C 5 min
Amplification (40 cycles)		
Secondary denaturation	94°C 15 s	94°C 15 s
Annealing (optics on)	58°C 30 s	55°C 30 s
Extension	72°C 30 s	72°C 30 s
Dissociation curve (1 cycle)		
Secondary denaturation	94°C 1 min	94°C 1 min
Annealing	58°C 1 min	55°C 1 min
Final denaturation	94°C 1 min	94°C 1 min

PCR=Polymerase chain reaction, GAPDH=Glyceraldehyde-3-phosphate dehydrogenase

### Cytology and histopathology of liver biopsy

Ultrasound-guided liver biopsy specimens were taken from the hepatic lesion using semi-automatic biopsy needle (Tru-cut needle; 18 gauge, 150 mm length). Specimens were rolled on the slide and stained by Diff-Quik (Harleco, Gibbstown, NJ) for cytological examination. The liver specimens were fixed 10% neutral buffered formalin, were routinely processed, paraffin embedded, and sectioned into 5 µm thick sections, and finally were stained with Hematoxylin and Eosin for histopathological examination [16].

### Statistical analysis

All quantitative data of hematology and serum biochemistry were presented as mean±standard error. The comparison between control and the diseased group was made using SPSS statistic program version 16.0; t-test. p≤0.05 was considered statistically significant.

## Results

### Clinical findings

**Figure-1:**
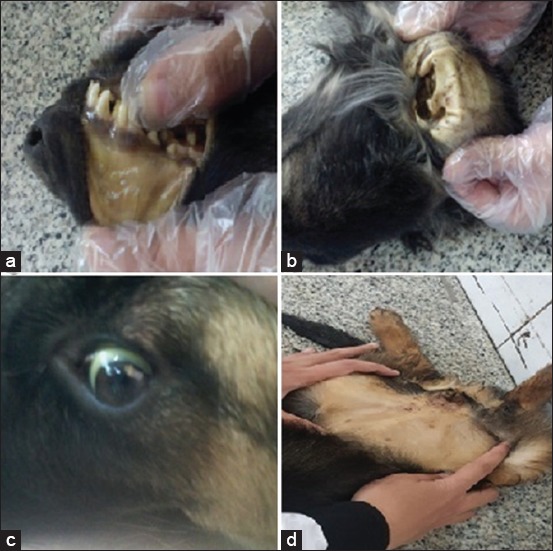
Clinical manifestations in Group A (acute hepatitis): (a) Icteric buccal mucous membrane. (b) Icteric inner aspect of the ear. (c) Icteric conjunctiva. (d) Icterus of ventral abdominal skin.

**Figure-2:**
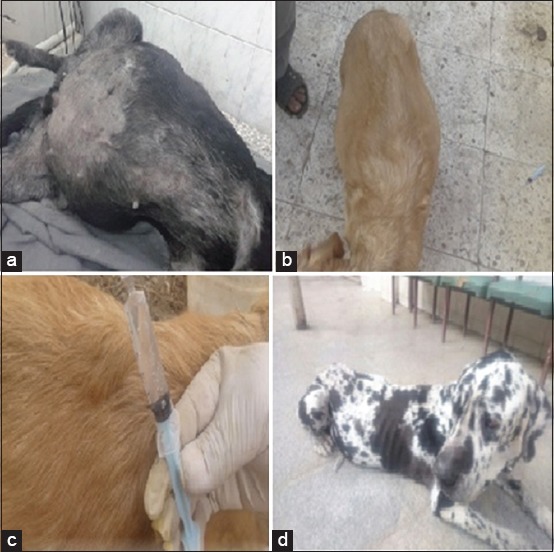
Clinical manifestations in Group B (chronic hepatitis): (a and b) Distension of abdomen with fluid (ascites). (c) Aspiration of abdominal fluid revealed clear transudate. (d) Severe weight loss.

Dogs with acute and chronic hepatitis showed no specific clinical signs including anorexia, depression, vomiting, diarrhea, and weight loss. Dogs with acute hepatitis showed characteristic clinical signs including icteric conjunctiva, buccal mucous membranes, inner aspect of the ear, and ventral abdominal skin (Figure-1). Dogs with chronic hepatitis showed characteristic clinical signs including fluid, abdominal distension and aspiration of this abdominal fluids revealed clear transudate (Figure-2).

### Abdominal ultrasonography

**Figure-3:**
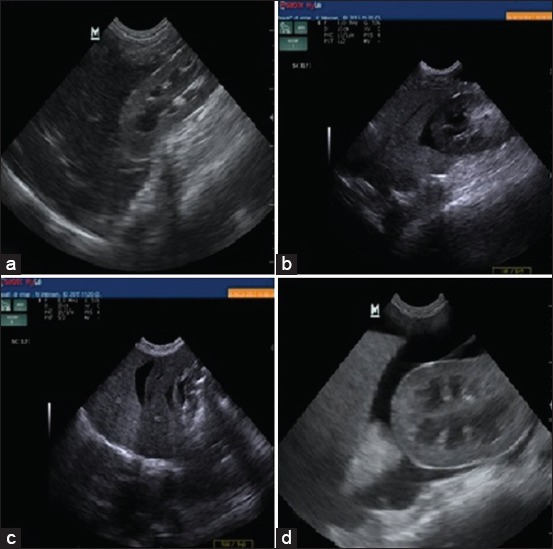
Ultrasonographic findings: (a) Decrease in hepatic echogenicity in comparison to renal cortex in dog with acute hepatitis. (b and d) Presence of clear anechoic ascites fluid separating right kidney and caudate liver lobe in dog with chronic hepatitis. (c) Increase in hepatic echogenicity than spleen echogenicity in dog with chronic hepatitis.

Dogs with acute hepatitis showed decreased hepatic echogenicity when compared to renal cortex with dilatation of hepatic blood vessels. Dogs with chronic hepatitis showed increased hepatic echogenicity, and a clear anechoic ascitic fluid of varied volume was seen in between liver lobes and separating right kidney from caudate liver lobe as shown in Figure-3.

### Hematobiochemical findings

Comparing the results of diseased dogs with control group, in Group A (acute hepatitis), red blood cell (RBC) count, packed cell volume (PCV) %, hemoglobin (HB) contents, platelet count, and neutrophils were significantly decreased with p≤0.019, p≤0.042, p≤0.05, p≤0.003, and p≤0.005, respectively, and significant increase in lymphocyte percentage (p≤0.004) was obtained. In Group B (chronic hepatitis), RBC count, PCV %, HB contents, and platelet count were significantly decreased with p≤0.028, p≤0.022, p≤0.008, and p≤0.017, respectively. Serum biochemical analysis of Group A showed significant increase of ALT, AST, ALP, and total bilirubin with p≤0.002, p≤0.008, p≤0.002, and p≤0.05, respectively, while Group B showed significant decrease in total protein and albumin with p≤0.006 and p≤0.007, respectively, with normal ALT, AST, ALP, and GGT as shown in Table-3.

**Table-3:** Hematology and biochemistry analysis.

Parameter/unit	Control group (n=10)	Group A (n=7)	Group B (n=9)
RBC count (106/c.mm)	6.98±0.27	4.46±0.63*	5.20±0.55*
PCV (%)	44.35±1.52	30.32±4.36*	30.70±3.93*
HB content (g/dl)	15.07±0.61	10.48±1.72*	10.72±1.03*
Platelet count (103/c.mm)	172.00±7.00	145.00±15.40*	131.20±10.38*
WBC count (103/c.mm	11.03±0.88	13.44±2.83	15.55±2.84
MCV (fl)	63.86±1.69	68.22±3.28	58.39±2.58
MCH (pg)	21.62±0.45	23.37±1.44	20.71±0.63
MCHC (g/dl)	33.95±0.58	34.53±1.95	35.66±1.56
Lymphocyte (%)	17.80±0.63	38.67±1.86*	19.40±6.19
Monocyte (%)	14.3±1.85	21.67±4.70	20.60±5.39
Eosinophils (%)	2.00±0.00	2.00±0.00	1.60±0.24
Basophils (%)	0.00±0.00	0.00±0.00	0.00±0.00
Staff (%)	1.33±0.33	2.67±0.66	1.60±0.600
Segmented no. (%)	65.22±0.90	35.00±2.89*	56.80±10.15
ALT (U/l)	21.79±2.20	105.40±11.55*	31.40±4.93
AST (U/l)	26.21±1.66	136.90±22.63*	30.74±1.04
ALP (U/l)	115.68±20.10	381.75±26.80*	153.86±21.20
GGT (U/l)	8.41±0.13	9.72±1.68	10.11±0.53
Total protein (g/dL)	5.89±0.35	4.92±0.04	3.23±0.52*
Albumin (g/dL)	2.59±0.07	2.30±0.31	1.78±0.19*
Total bilirubin (mg/dL)	0.11±0.03	3.18±1.22*	0.48±0.26

* Data are presented as mean±standard error, P≤0.05, RBC=Red blood cells, HB=Hemoglobin, WBCs=White blood cells, MCV=Mean corpuscular volume, MCH=Mean corpuscular hemoglobin, MCHC=Mean corpuscular hemoglobin concentration, ALT=Alanine aminotransferase, AST=Aspartate aminotransferase, ALP=Alkaline phosphatase, GGT=Gamma-glutamyltransferase

### Serum hepatocyte-derived miRNA-122

In comparison with the control group, the serum hepatocyte-derived miRNA-122 values revealed significant increases as 6.23-fold in Group A (acute hepatitis) with p≤0.001 and 6.27-fold in Group B (chronic hepatitis) with p≤0.0.001 as shown in Table-4.

**Table-4:** Serum hepatocyte-derived miRNA-122 analysis.

Group	GAPDH CT	MiR-122 CT	Δ CT	Δ Δ CT	Fold change (2-Δ Δ CT)
Control group	25.54	23.44	2.1	0	1
Group A	24.50	19.76	4.74	2.64	6.233
Group B	24.81	20.06	4.75	2.65	6.276

miRNA = microRNAs, GAPDH = Glyceraldehyde-3-phosphate dehydrogenase, CT = Cycle threshold

### The cytological and histopathological findings of liver biopsy in Group A (acute hepatitis)

**Figure-4:**
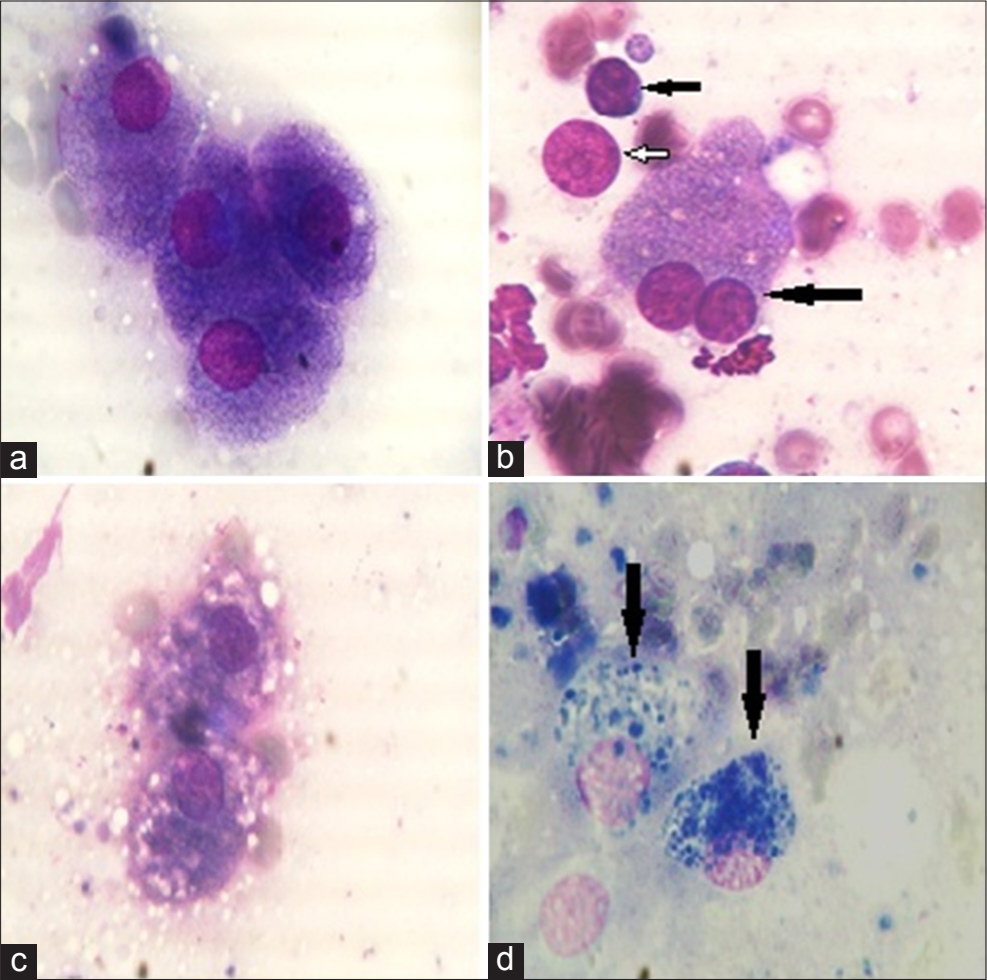
Cytological findings in all groups: (a) Normal hepatocytes of control healthy group. Cells contain centrally located nuclei with basophilic cytoplasm and a single prominent pale-blue nucleolus (arrow) (field stain 1000×). (b) Hepatic smears of acute hepatitis group. Binucleated hepatocytes (long arrow) with the presence of lymphocyte (short arrow), naked (free) nuclei with prominent nucleoli (white arrow) (field stain 1000×). (c) Moderate vacuolar changes in hepatocytes of acute hepatitis group (field stain 1000×). (d) Two hepatocytes with accumulation of bile pigment (arrow) (field stain 1000×).

**Figure-5:**
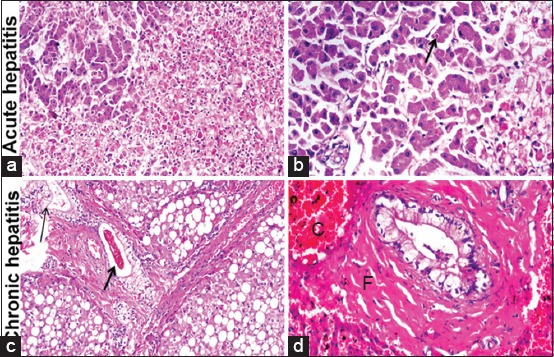
Histopathology findings in canine acute and chronic hepatitis groups: (a) Liver showing diffuse hepatocellular disarrangement, massive hepatocellular necrosis with inflammatory cells infiltration (200×). (b) Liver showing apoptosis of individual hepatocytes (arrow) (400×). (c) Liver showing portal fibrosis with extension of fibrous tissue into hepatic lobules associated with cholestasis (thick arrow), bile duct hyperplasia (thin arrow), and diffuse macrovesicular steatosis of hepatocytes (200×). (d) Liver showing periductular fibrosis (F), portal congestion (C), note the vacuolization of biliary epithelium (400×).

Cytological examination of hepatic smears of the control healthy group consisted largely of uniform, large, or slightly oval shape hepatocytes with basophilic cytoplasm. Nuclei were round, centrally placed with coarse chromatin and a single prominent nucleolus (Figure-4a). Cytological examination of acute hepatitis group showed large numbers of binucleated hepatocytes (Figure-4b) with the presence of large numbers of lymphocytes, Kupffer cells and neutrophils. Naked (free) nuclei with prominent nucleoli were metastasized all over the hepatic smears (Figure-4b). Moreover, cytological examination of acute hepatitis group showed moderate vacuolar changes in hepatocytes (Figure-4c) with accumulation of brownish-green pigment inside some individual hepatocytes (Figure-4d). Microscopic examination of hepatic biopsy of acute hepatitis group revealed various histopathological alterations, necrosis of hepatocytes was evident and unique, the lesion characterized by random distribution of necrosis referred to single cell necrosis, and inflammatory cell infiltrations including few neutrophils, macrophages, and lymphocytes were evident. Apoptosis of individual hepatocytes was detected (Figure-5a and b).

### Histopathological findings of liver tissue in Group B (chronic hepatitis)

Marked histopathological alterations were evident in chronic hepatitis group. The lesions included massive portal fibrosis that extends into hepatic parenchyma. There was periductular proliferation with mononuclear cell infiltration; the hepatocytes showed diffuse macrovesicular steatosis. Portal and sinusoidal congestion were detected (Figure-5c and d).

## Discussion

Liver diseases are difficult to diagnose as dogs remain subclinical for a long period. In addition, these subclinical dogs often show normal enzymatic levels so that they are easily missed by current screening methods [5]. For early diagnosis, new hepatic diagnostic biomarkers are needed to overcome this lack in the sensitivity of current screening methods [6]. In the present study, the diagnostic value of hepatocyte-derived miRNA-122 in dogs with acute and chronic hepatitis was investigated. Hepatocyte-derived miRNA-122 is emerging as a biomarker for hepatic disease because they play critical roles in liver development and metabolism and is measurable in blood even after prolonged storage [17].

Clinically, Jaundice noticed in dogs with acute hepatitis may be related to the impairment of bilirubin clearance by the liver. Vomiting may be related to decrease the ability of liver to clear toxins which stimulate vomiting center [18]. On the other hand, ascites noticed in dogs with chronic hepatitis may be related to intrahepatic portal hypertension and diminished albumin assembly [19], weight loss may be attributed to anorexia and catabolic state, and melena may be as a result of coagulopathies.

Ultrasonographically, decreased hepatic echogenicity in Group A (acute hepatitis) may be attributed to congestion of the liver [20] and increased hepatic echogenicity in Group B (chronic hepatitis) may be attributed to fibrosis of liver tissue [19].

Normocytic normochromic anemia in the hemogram of both groups may be related to high levels of bile acid causing fragility of RBCs [21,22]. Thrombocytopenia also an abnormality revealed by CBC which related to the reduction of thrombopoietin formation by the liver along with elevated utilization of thrombocytes [23].

Hepatic enzymes are the most sensitive biomarkers used for evaluating the function and integrity of liver cells. Thus, the presence of such enzymes within the circulation is considered clear evidence for the damage of hepatocytes cell membrane. In the present study, significant elevation of serum hepatic enzymes (ALT and AST) in acute hepatitis group [24,25] gives evidence for a hepatic injury that confirmed cytologically by vacuolar degeneration, a large number of binucleated hepatocytes, and presence of mixed inflammatory cells with free nuclei as a sign of tissue irritation. Bile formation is a secretory function of the liver which appears cytologically as intracellular brown-greenish pigment comes in correlation with significant elevation of serum ALP and serum total bilirubin in acute hepatitis group. In contrast, chronic hepatitis group showed a significant decrease in total protein and albumin indicating hepatic dysfunction and albumin synthesis is decreased by the liver [26] causing hypoproteinemia. In addition, ALT and AST levels were located in normal range due to fibrosis of the most normal hepatocyte which has a direct effect on these findings [27].

The study showed a significant elevation of serum hepatocyte-derived miRNA-122 in dogs with acute and chronic hepatitis. These findings come in accordance with human studies where serum miRNA-122 level elevated due to hepatocellular injury of different causes [9,10,28,29] and veterinary study where serum miRNA-122 level elevated in acute and chronic hepatitis [30]; furthermore, it confirms data from a West European Labrador Retriever population in a completely unrelated array of breeds [12]. The most important finding in this study is the elevation of serum miR-122 in dogs suffering from chronic hepatitis with normal ALT level which commonly used for the detection of hepatocellular injury which suggests that miR-122 is more sensitive and stable indicator for the detection of hepatic injury than ALT. These findings come in accordance with human research compared level of miRNA-122 to ALT in human chronically infected with hepatitis C; serum miRNA-122 level was highly elevated even in patients with normal aminotransferases [10].

Liver biopsy often required to establish a definitive diagnosis in dogs with hepatobiliary disease. Necrosis was evident in Group A (acute hepatitis) and was referred to single cell necrosis rather than zonal coagulative necrosis as the random distribution of necrotic cells; in addition, the appearance of circumscribed eosinophilic body was observed in the study and was previously described in canine viral hepatitis [31]. In contrast, in Group B (chronic hepatitis), the histopathology of liver tissue revealed massive fibrous tissue proliferation in hepatic parenchyma and periductular proliferation and mononuclear cell infiltration with portal congestion [32].

## Conclusion

The study suggests that serum hepatocyte-derived miRNA-122 can be used as non-invasive, easily measurable, and stable diagnostic blood biomarker for the diagnosis of acute and chronic hepatitis of dogs and provides a valuable addition to the routine methods of hepatobiliary disease diagnosis. miRNA-122 is more sensitive and diagnostic in dogs suffering from chronic hepatitis than ALT.

## Authors’ Contributions

SRE collected materials of the study and was responsible for laboratory work. AAK designed the study, provided guidance during the entire study, and corrected the manuscript. TAB helped in the preparation of the manuscript and data analysis. FAT was responsible for ultrasonographic examination and interpretation. ISS was responsible for cytology of liver biopsy. FFM was responsible for the histopathology of the liver biopsy. All authors have read and approved the final version of the manuscript.
